# *N*′-(5-Bromo-2-methoxy­benzyl­idene)-3,4-methyl­enedioxy­benzohydrazide

**DOI:** 10.1107/S1600536809022818

**Published:** 2009-06-20

**Authors:** Ya-Li Sang, Xue-Song Lin

**Affiliations:** a Department of Chemistry, Chifeng University, Chifeng 024001, People’s Republic of China

## Abstract

In the title mol­ecule, C_16_H_13_BrN_2_O_4_, the two benzene rings form a dihedral angle of 74.9 (2)°. In the crystal, mol­ecules are linked *via* inter­molecular N—H⋯O hydrogen bonds into chains propagating along the *c* axis.

## Related literature

For the biological activity of hydrazone derivatives, see: Khattab (2005 ▶); Küçükgüzel *et al.* (2003 ▶); Cukurovali *et al.* (2006 ▶). For the crystal structures of related compounds, see: Fun *et al.* (2008 ▶); Wei *et al.* (2009 ▶); Khaledi *et al.* (2008 ▶); Yang *et al.* (2008 ▶).
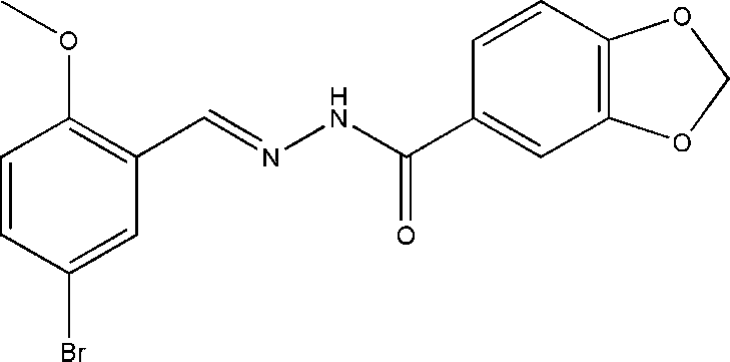


## Experimental

### 

#### Crystal data

C_16_H_13_BrN_2_O_4_
*M* *_r_* = 377.19
Monoclinic, 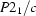

*a* = 12.678 (1) Å
*b* = 16.217 (2) Å
*c* = 7.846 (2) Å
β = 104.804 (3)°
*V* = 1559.6 (5) Å^3^
*Z* = 4
Mo *K*α radiation
μ = 2.66 mm^−1^
*T* = 298 K
0.30 × 0.28 × 0.27 mm

#### Data collection

Bruker SMART CCD area-detector diffractometer
Absorption correction: multi-scan (*SADABS*; Sheldrick, 1996 ▶) *T*_min_ = 0.503, *T*_max_ = 0.534 (expected range = 0.460–0.488)
8368 measured reflections
3110 independent reflections
1932 reflections with *I* > 2σ(*I*)
*R*_int_ = 0.032

#### Refinement

*R*[*F* ^2^ > 2σ(*F* ^2^)] = 0.039
*wR*(*F* ^2^) = 0.096
*S* = 1.04
3110 reflections
212 parameters
1 restraint
H atoms treated by a mixture of independent and constrained refinement
Δρ_max_ = 0.26 e Å^−3^
Δρ_min_ = −0.47 e Å^−3^

### 

Data collection: *SMART* (Bruker, 2002 ▶); cell refinement: *SAINT* (Bruker, 2002 ▶); data reduction: *SAINT*; program(s) used to solve structure: *SHELXS97* (Sheldrick, 2008 ▶); program(s) used to refine structure: *SHELXL97* (Sheldrick, 2008 ▶); molecular graphics: *SHELXTL* (Sheldrick, 2008 ▶); software used to prepare material for publication: *SHELXL97*.

## Supplementary Material

Crystal structure: contains datablocks global, I. DOI: 10.1107/S1600536809022818/cv2572sup1.cif

Structure factors: contains datablocks I. DOI: 10.1107/S1600536809022818/cv2572Isup2.hkl

Additional supplementary materials:  crystallographic information; 3D view; checkCIF report

## Figures and Tables

**Table 1:** Hydrogen-bond geometry (Å, °)

*D*—H⋯*A*	*D*—H	H⋯*A*	*D*⋯*A*	*D*—H⋯*A*
N2—H2⋯O2^i^	0.89 (3)	1.96 (3)	2.841 (3)	168 (3)

Symmetry code: (i) 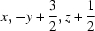.

**Fig. 1.**
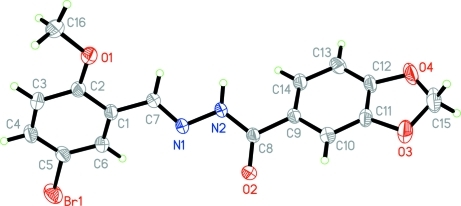
The molecular structures of the title compound, showing 30% probability displacement ellipsoids and the atom-numbering scheme.

## supplementary crystallographic information

### Comment

Hydrazone compounds have been widely investigated due to their interesting biological properties, such as antibacterial and antitumor activities (Khattab, 2005; Küçükgüzel *et al.*, 2003; Cukurovali *et al.*, 2006). Recently, a number of crystal structures of hydrazone derivatives have been reported (Fun *et al.*, 2008; Wei *et al.*, 2009; Khaledi *et al.*, 2008; Yang *et al.*, 2008). In this paper, the crystal structure of the title new hydrazone compound is reported.

The molecular structure of the title compound is shown inFig. 1. The molecule adopts an *E* configuration with respect to the C═N bond. The dihedral angle between the two substituted benzene rings is 74.9 (2)°.

In the crystal, the molecules are linked *via* intermolecular N—H···O hydrogen bonds (Table 1) into chains propagated along *c* axis.

### Experimental

3,4-(Methylenedioxy)benzohydrazide (1.0 mmol, 180.2 mg) and 5-bromo-2-methoxybenzaldehyde (1.0 mmol, 215.0 mg) were mixed and refluxed in ethanol (50 ml). The mixture was stirred for 1 h to give a clear colorless solution. Colourless crystals of the title compound were formed by slow evaporation of the solution in air.

### Refinement

Atom H2 attached to N2 was located in a difference map and refined with N–H distance restraint of 0.90 (3) Å. The other H atoms were positioned geometrically [*d*(C–H) = 0.93–0.97 Å], and refined using a riding model, with *U*_iso_(H) = 1.2*U*_eq_(C).

### Crystal data

**Table:**

C_16_H_13_BrN_2_O_4_	*F*(000) = 760
*M**_r_* = 377.19	*D*_x_ = 1.606 Mg m^−^^3^
Monoclinic, *P*2_1_/*c*	Mo *K*α radiation, λ = 0.71073 Å
Hall symbol: -P 2ybc	Cell parameters from 2058 reflections
*a* = 12.678 (1) Å	θ = 2.5–24.5°
*b* = 16.217 (2) Å	µ = 2.66 mm^−^^1^
*c* = 7.846 (2) Å	*T* = 298 K
β = 104.804 (3)°	Block, colourless
*V* = 1559.6 (5) Å^3^	0.30 × 0.28 × 0.27 mm
*Z* = 4	

### Data collection

**Table:**

Bruker SMART CCD area-detector diffractometer	3110 independent reflections
Radiation source: fine-focus sealed tube	1932 reflections with *I* > 2σ(*I*)
graphite	*R*_int_ = 0.032
ω scans	θ_max_ = 26.2°, θ_min_ = 1.7°
Absorption correction: multi-scan (*SADABS*; Sheldrick, 1996)	*h* = −12→15
*T*_min_ = 0.503, *T*_max_ = 0.534	*k* = −19→19
8368 measured reflections	*l* = −9→4

### Refinement

**Table:**

Refinement on *F*^2^	Primary atom site location: structure-invariant direct methods
Least-squares matrix: full	Secondary atom site location: difference Fourier map
*R*[*F*^2^ > 2σ(*F*^2^)] = 0.039	Hydrogen site location: inferred from neighbouring sites
*wR*(*F*^2^) = 0.096	H atoms treated by a mixture of independent and constrained refinement
*S* = 1.04	*w* = 1/[σ^2^(*F*_o_^2^) + (0.0397*P*)^2^ + 0.3363*P*] where *P* = (*F*_o_^2^ + 2*F*_c_^2^)/3
3110 reflections	(Δ/σ)_max_ < 0.001
212 parameters	Δρ_max_ = 0.26 e Å^−^^3^
1 restraint	Δρ_min_ = −0.47 e Å^−^^3^

### Special details

**Table:**

Geometry. All e.s.d.'s (except the e.s.d. in the dihedral angle between two l.s. planes) are estimated using the full covariance matrix. The cell e.s.d.'s are taken into account individually in the estimation of e.s.d.'s in distances, angles and torsion angles; correlations between e.s.d.'s in cell parameters are only used when they are defined by crystal symmetry. An approximate (isotropic) treatment of cell e.s.d.'s is used for estimating e.s.d.'s involving l.s. planes.
Refinement. Refinement of *F*^2^ against ALL reflections. The weighted *R*-factor *wR* and goodness of fit *S* are based on *F*^2^, conventional *R*-factors *R* are based on *F*, with *F* set to zero for negative *F*^2^. The threshold expression of *F*^2^ > σ(*F*^2^) is used only for calculating *R*-factors(gt) *etc*. and is not relevant to the choice of reflections for refinement. *R*-factors based on *F*^2^ are statistically about twice as large as those based on *F*, and *R*- factors based on ALL data will be even larger.

### Fractional atomic coordinates and isotropic or equivalent isotropic displacement parameters (Å^2^)

**Table:**

	*x*	*y*	*z*	*U*_iso_*/*U*_eq_	
Br1	0.79365 (3)	0.77734 (2)	0.01187 (6)	0.08050 (19)	
O1	0.47896 (16)	0.98772 (12)	0.2718 (3)	0.0551 (6)	
O2	0.23491 (16)	0.66086 (12)	−0.0682 (3)	0.0495 (5)	
O3	−0.13891 (18)	0.56492 (16)	0.0073 (3)	0.0777 (7)	
O4	−0.1167 (2)	0.54766 (14)	0.3048 (4)	0.0750 (7)	
N1	0.38458 (18)	0.76267 (14)	0.1294 (3)	0.0420 (6)	
N2	0.29501 (19)	0.73440 (14)	0.1821 (3)	0.0427 (6)	
C1	0.5282 (2)	0.86061 (18)	0.1769 (4)	0.0426 (7)	
C2	0.5525 (2)	0.94368 (18)	0.2108 (4)	0.0434 (7)	
C3	0.6454 (2)	0.9767 (2)	0.1762 (4)	0.0542 (8)	
H3	0.6603	1.0327	0.1944	0.065*	
C4	0.7157 (3)	0.9274 (2)	0.1152 (4)	0.0561 (8)	
H4	0.7787	0.9498	0.0939	0.067*	
C5	0.6934 (2)	0.8455 (2)	0.0857 (4)	0.0504 (8)	
C6	0.6000 (2)	0.81226 (19)	0.1145 (4)	0.0482 (8)	
H6	0.5848	0.7566	0.0918	0.058*	
C7	0.4292 (2)	0.82617 (17)	0.2101 (4)	0.0423 (7)	
H7	0.3982	0.8514	0.2922	0.051*	
C8	0.2255 (2)	0.68194 (17)	0.0780 (4)	0.0392 (7)	
C9	0.1363 (2)	0.64994 (16)	0.1497 (4)	0.0382 (7)	
C10	0.0401 (2)	0.62571 (18)	0.0293 (4)	0.0495 (8)	
H10	0.0306	0.6312	−0.0917	0.059*	
C11	−0.0387 (2)	0.59384 (18)	0.0980 (5)	0.0490 (8)	
C12	−0.0262 (2)	0.58422 (18)	0.2737 (5)	0.0521 (8)	
C13	0.0667 (3)	0.6066 (2)	0.3945 (4)	0.0583 (9)	
H13	0.0750	0.5994	0.5148	0.070*	
C14	0.1485 (2)	0.64061 (18)	0.3280 (4)	0.0480 (7)	
H14	0.2131	0.6576	0.4060	0.058*	
C15	−0.1939 (3)	0.5446 (2)	0.1383 (6)	0.0819 (12)	
H15A	−0.2524	0.5835	0.1351	0.098*	
H15B	−0.2250	0.4897	0.1173	0.098*	
C16	0.5016 (3)	1.07233 (19)	0.3115 (4)	0.0610 (9)	
H16A	0.5691	1.0773	0.4005	0.092*	
H16B	0.4437	1.0961	0.3538	0.092*	
H16C	0.5073	1.1008	0.2070	0.092*	
H2	0.278 (3)	0.7609 (18)	0.271 (3)	0.080*	

### Atomic displacement parameters (Å^2^)

**Table:**

	*U*^11^	*U*^22^	*U*^33^	*U*^12^	*U*^13^	*U*^23^
Br1	0.0737 (3)	0.0813 (3)	0.1027 (3)	0.0049 (2)	0.0521 (2)	−0.0014 (2)
O1	0.0564 (13)	0.0467 (13)	0.0667 (14)	−0.0076 (10)	0.0240 (12)	−0.0086 (11)
O2	0.0570 (13)	0.0515 (12)	0.0467 (12)	−0.0134 (10)	0.0255 (11)	−0.0098 (10)
O3	0.0461 (14)	0.0882 (18)	0.098 (2)	−0.0217 (13)	0.0169 (14)	−0.0130 (15)
O4	0.0626 (15)	0.0723 (17)	0.105 (2)	−0.0207 (13)	0.0492 (16)	0.0006 (15)
N1	0.0403 (13)	0.0441 (15)	0.0456 (15)	−0.0083 (11)	0.0186 (12)	−0.0008 (12)
N2	0.0427 (14)	0.0452 (14)	0.0465 (15)	−0.0125 (12)	0.0226 (12)	−0.0047 (12)
C1	0.0418 (17)	0.0469 (18)	0.0389 (17)	−0.0100 (14)	0.0097 (14)	0.0029 (14)
C2	0.0441 (17)	0.0469 (18)	0.0390 (17)	−0.0061 (14)	0.0104 (14)	0.0006 (14)
C3	0.0524 (19)	0.0492 (19)	0.062 (2)	−0.0178 (15)	0.0176 (17)	−0.0060 (16)
C4	0.0467 (18)	0.064 (2)	0.061 (2)	−0.0142 (16)	0.0199 (17)	0.0026 (18)
C5	0.0470 (18)	0.056 (2)	0.0521 (19)	−0.0026 (15)	0.0204 (16)	0.0014 (16)
C6	0.0516 (18)	0.0436 (17)	0.0513 (19)	−0.0060 (15)	0.0165 (16)	0.0023 (15)
C7	0.0441 (17)	0.0437 (18)	0.0420 (17)	−0.0062 (14)	0.0164 (14)	−0.0020 (14)
C8	0.0405 (16)	0.0356 (16)	0.0446 (17)	−0.0027 (13)	0.0168 (14)	0.0003 (14)
C9	0.0357 (15)	0.0357 (15)	0.0455 (17)	−0.0015 (12)	0.0148 (14)	0.0006 (14)
C10	0.0450 (18)	0.055 (2)	0.0494 (19)	−0.0050 (15)	0.0141 (16)	−0.0023 (16)
C11	0.0330 (16)	0.0450 (18)	0.069 (2)	−0.0062 (14)	0.0126 (16)	−0.0056 (16)
C12	0.0466 (19)	0.0402 (17)	0.079 (2)	−0.0097 (15)	0.0341 (18)	−0.0024 (17)
C13	0.070 (2)	0.062 (2)	0.052 (2)	−0.0107 (18)	0.0309 (19)	0.0063 (17)
C14	0.0442 (17)	0.0503 (19)	0.0515 (19)	−0.0075 (14)	0.0158 (15)	0.0019 (15)
C15	0.048 (2)	0.075 (3)	0.128 (4)	−0.0163 (19)	0.031 (3)	−0.006 (3)
C16	0.072 (2)	0.047 (2)	0.067 (2)	−0.0021 (17)	0.0240 (19)	−0.0059 (17)

### Geometric parameters (Å, °)

**Table:**

Br1—C5	1.884 (3)	C4—H4	0.9300
O1—C2	1.355 (3)	C5—C6	1.371 (4)
O1—C16	1.420 (4)	C6—H6	0.9300
O2—C8	1.232 (3)	C7—H7	0.9300
O3—C11	1.370 (3)	C8—C9	1.479 (4)
O3—C15	1.420 (4)	C9—C14	1.376 (4)
O4—C12	1.367 (3)	C9—C10	1.395 (4)
O4—C15	1.419 (5)	C10—C11	1.353 (4)
N1—C7	1.264 (3)	C10—H10	0.9300
N1—N2	1.382 (3)	C11—C12	1.356 (4)
N2—C8	1.341 (4)	C12—C13	1.359 (4)
N2—H2	0.89 (3)	C13—C14	1.389 (4)
C1—C6	1.382 (4)	C13—H13	0.9300
C1—C2	1.392 (4)	C14—H14	0.9300
C1—C7	1.458 (4)	C15—H15A	0.9700
C2—C3	1.383 (4)	C15—H15B	0.9700
C3—C4	1.372 (4)	C16—H16A	0.9600
C3—H3	0.9300	C16—H16B	0.9600
C4—C5	1.366 (4)	C16—H16C	0.9600
			
C2—O1—C16	117.9 (2)	C14—C9—C10	120.5 (3)
C11—O3—C15	105.4 (3)	C14—C9—C8	121.8 (3)
C12—O4—C15	105.3 (3)	C10—C9—C8	117.6 (3)
C7—N1—N2	114.6 (2)	C11—C10—C9	116.4 (3)
C8—N2—N1	119.4 (2)	C11—C10—H10	121.8
C8—N2—H2	122 (2)	C9—C10—H10	121.8
N1—N2—H2	117 (2)	C10—C11—C12	122.9 (3)
C6—C1—C2	118.9 (3)	C10—C11—O3	127.2 (3)
C6—C1—C7	121.4 (3)	C12—C11—O3	109.9 (3)
C2—C1—C7	119.6 (3)	C11—C12—C13	122.2 (3)
O1—C2—C3	124.2 (3)	C11—C12—O4	110.2 (3)
O1—C2—C1	116.1 (2)	C13—C12—O4	127.5 (3)
C3—C2—C1	119.7 (3)	C12—C13—C14	116.2 (3)
C4—C3—C2	120.3 (3)	C12—C13—H13	121.9
C4—C3—H3	119.8	C14—C13—H13	121.9
C2—C3—H3	119.8	C9—C14—C13	121.7 (3)
C5—C4—C3	120.1 (3)	C9—C14—H14	119.2
C5—C4—H4	119.9	C13—C14—H14	119.2
C3—C4—H4	119.9	O4—C15—O3	107.9 (3)
C4—C5—C6	120.3 (3)	O4—C15—H15A	110.1
C4—C5—Br1	119.7 (2)	O3—C15—H15A	110.1
C6—C5—Br1	119.9 (3)	O4—C15—H15B	110.1
C5—C6—C1	120.7 (3)	O3—C15—H15B	110.1
C5—C6—H6	119.7	H15A—C15—H15B	108.4
C1—C6—H6	119.7	O1—C16—H16A	109.5
N1—C7—C1	121.2 (3)	O1—C16—H16B	109.5
N1—C7—H7	119.4	H16A—C16—H16B	109.5
C1—C7—H7	119.4	O1—C16—H16C	109.5
O2—C8—N2	122.5 (2)	H16A—C16—H16C	109.5
O2—C8—C9	121.5 (3)	H16B—C16—H16C	109.5
N2—C8—C9	116.0 (2)		

### Hydrogen-bond geometry (Å, °)

**Table:**

*D*—H···*A*	*D*—H	H···*A*	*D*···*A*	*D*—H···*A*
N2—H2···O2^i^	0.89 (3)	1.96 (3)	2.841 (3)	168 (3)

Symmetry codes: (i) *x*, −*y*+3/2, *z*+1/2.
